# Zerlegung des Arbeitsschutzes in der Fleischindustrie durch Werkverträge – und die Notwendigkeit integrativen Arbeitsschutzes

**DOI:** 10.1007/s41449-020-00232-9

**Published:** 2020-11-20

**Authors:** Wolfhard Kohte, Cathleen Rabe-Rosendahl

**Affiliations:** Martin-Luther-Universität Halle-Wittenberg, Universitätsplatz 5, 06108 Halle (Saale), Deutschland

**Keywords:** Fleischwirtschaft, Arbeitsschutz, Corona, Arbeitsschutzkontrollgesetz, Hygienerecht, Lebensmittelhygiene, Meat industry, Corona, Occupational health and safety law, Food hygiene legislation

## Abstract

Die katastrophalen Arbeitsbedingungen in der Fleischwirtschaft sind nicht erst seit der Corona-Pandemie bekannt, jedoch erwiesen sich in diesem Frühjahr insbesondere Schlachthöfe als Hotspots der Sars-CoV-2-Verbreitung. Die Bundesregierung hat im Sommer dieses Jahres einen Entwurf für ein Arbeitsschutzkontrollgesetz vorgelegt. Der Gesetzesentwurf sieht unter anderem ein Verbot des Einsatzes von Werkverträgen und Leiharbeit im Kernbereich der Fleischwirtschaft ab 1. Januar bzw 1. April 2021 vor.

Der vorliegende Artikel zeichnet die Versäumnisse der letzten Jahre nach und zeigt, warum in Fremdbeschäftigungskonstellationen die wichtige zwischenbetriebliche Kooperation im Arbeitsschutz in der Fleischindustrie kein Erfolgsmodell darstellen kann, da sich in der Fleischindustrie die Werkunternehmen in Bezug auf den Arbeitsschutz nicht durchsetzungsfähig und –willig sind. Der Beitrag beleuchtet, warum die Organisationspflichten des Betriebsinhabers ausschlaggebend für die Einhaltung nationaler und unionsrechtlicher Arbeitsschutz- und Lebensmittelhygienevorschriften ist und letztlich durch die Arbeitsschutzaufsichtsbehörden kontrolliert werden müssen. Der Beitrag unterstützt die Ziele des Gesetzentwurfs, doch ist zum Zeitpunkt des Redaktionsschlusses (16.11.2020) noch offen, ob der Entwurf ohne substantielle Verschlechterungen im Bundestag beschlossen wird.

## Abstract (en)

The devastating working conditions in the meat industry have not only been known since the Corona pandemic, but this spring slaughterhouses in particular proved to be hotspots of the Sars-CoV-2 spread. In the summer of this year, the Federal Government presented a draft for an occupational health and safety control law. Among other things, the bill provides for a ban on the use of contracts to produce a work (Werkverträge) and temporary agency work in the core area of the meat industry from 1 January and 1 April 2021 respectively.

This article traces the omissions of recent years and shows why, in third-party employment constellations, the important inter-company cooperation in occupational health and safety cannot represent a model for success in the meat industry, since in the meat industry the contracting companies are neither able nor willing to assert themselves with regard to occupational health and safety. The article highlights why the proprietor’s organisational obligations are crucial for compliance with national and EU occupational health and safety law as well as food hygiene regulations, and must ultimately be monitored by the occupational health and safety inspectorates. The contribution supports the objectives of the bill, but at the time of the editorial deadline (16.11.2020) it is still uncertain whether the Bundestag will pass the law without substantial deterioration.

## Einleitung

Nicht erst seit dem sprunghaften Anstieg von Corona-Infektionen in Schlachthöfen des Tönnies-Konzerns stehen die Arbeitsbedingungen in der Fleischindustrie, insbesondere der unzureichende Arbeitsschutz und die damit verbundene hohe Anzahl von Arbeitsunfällen, sowie die oftmals alarmierende Unterkunftssituation^1^ wieder in der Kritik. Wie die Bundesregierung ihrem Gesetzesentwurf für ein Gesetz zur Verbesserung des Vollzugs im Arbeitsschutz (Arbeitsschutzkontrollgesetz) zutreffend voranstellt, ist eine Kernaufgabe staatlichen Handelns, Rahmenbedingungen für gesunde, sichere und menschengerecht gestaltete Arbeitsbedingungen der Beschäftigten zu schaffen^2^. In der Fleischindustrie ist dies trotz einiger gesetzgeberischer Bemühungen^3^ und freiwilliger Selbstverpflichtungen der Branche in den letzten Jahren nicht gelungen – obwohl, wie zu zeigen sein wird, die Rechtslage, insbesondere im Hinblick auf Arbeitsschutz und Lebensmittelhygiene, bereits zuvor eindeutige Maßnahmen erforderte.

## Unzureichender Arbeitsschutz in der Fleischindustrie – ein lange bekanntes Problem^4^

### Untersuchungen der Arbeitsschutzverwaltung NRW aus 2013

Bereits 2013 gab es eine Untersuchung der Arbeitsschutzverwaltung Nordrhein-Westfalen, welche schon damals ergab, dass in der Fleischindustrie eklatante Mängel im Arbeitsschutz zu verzeichnen sind. Zu diesem Zeitpunkt gab es bereits einen überproportional hohen Anteil von Fremdfirmen in der Fleischindustrie,^5^ welche oft Beschäftigte aus mittel- und osteuropäischen EU-Mitgliedsstaaten einsetzten. Eigene Beschäftigte waren oftmals nur noch im Verwaltungsbereich der Unternehmen tätig. Der mangelhafte Arbeitsschutz steht hierbei in einem klaren Zusammenhang mit dem Fremdfirmeneinsatz: Bei 79 % der Betriebe, die Werkunternehmer einsetzten, erfolgte keine oder nur eine unzureichende Abstimmung zwischen dem Fleischbetrieb und dem Werkvertragsunternehmen; bei den Werkvertragsunternehmen gab es bei über zwei Drittel Mängel in der Gefährdungsbeurteilung; bei fast der Hälfte der Zerlegebetriebe fehlten schriftlichen Betriebsanweisungen in der Sprache der Beschäftigten (z. B. über die Verwendung von Gefahrstoffen).^6^ In keiner der überprüften Werkvertragsfirmen gab es einen Betriebsrat.

2014 hat der Gesetzgeber die Fleischindustrie in das Arbeitnehmerentsende-Gesetz aufgenommen,^7^ so dass eine Erstreckung des Branchenmindestlohns auch auf entsandte Arbeitnehmer möglich wurde (Allgemeinverbindlichkeitserklärung nach § 4 AEntG). Kurz zuvor hatten sich die Tarifvertragsparteien der Fleischbranche auf einen Mindestlohntarifvertrag geeinigt. 2015 unterzeichneten in Kooperation mit dem Bundesministerium für Wirtschaft und Energie die größten Unternehmen der Fleischwirtschaft eine Selbstverpflichtung für attraktivere Arbeitsbedingungen, insbesondere Einhaltung des Arbeitszeitrechts und des Rechts der Entgeltfortzahlung. Spätere Untersuchungen zeigten, dass diese Erklärung nicht zu einer effektiven Verbesserung des Arbeitsschutzes geführt hatte^8^.

### Das Gesetz zur „Sicherung von Arbeitnehmerrechten in der Fleischwirtschaft“ (GSA-Fleisch) von 2017

Das Gesetz zur „Sicherung von Arbeitnehmerrechten in der Fleischwirtschaft“ (GSA-Fleisch) passierte das Parlament einigermaßen überraschend in einem sogenannten Omnibus-Gesetz.^9^ Ziele des Gesetzes sind die Sicherung von Rechten und Ansprüchen der Arbeitnehmerinnen und Arbeitnehmer sowie die Verhinderung von Umgehungen der Pflicht zur Zahlung von Sozialversicherungsbeiträgen durch die Beauftragung von Nachunternehmern (§ 1 GSA). § 2 definiert den Geltungsbereich als „Fleischwirtschaft“ und verweist hierbei auf die Definition im Entsenderecht (§ 6 Abs. 10 des damaligen AEntG^10^). Hauptanliegen des Gesetzes war die Ausdehnung der bisher nur für Unternehmer des Baugewerbes, die andere Unternehmer mit der Erbringung von Bauleistungen beauftragen, geltende System der Haftung für Gesamtsozialversicherungsbeiträge und für Beiträge zur gesetzlichen Unfallversicherung auf den Bereich der Fleischwirtschaft. So sollte die Umgehung der Pflicht zur ordnungsgemäßen Zahlung von Sozialversicherungsbeiträgen durch die im Bereich der Fleischwirtschaft – ebenso wie im Baugewerbe – verbreitete Beauftragung von Nachunternehmern verhindert werden. Darüber hinaus regelt das GSA Fleisch explizit die Pflicht für den Arbeitgeber, Arbeitnehmerinnen und Arbeitnehmern Arbeitsmittel, die aus Hygienegründen oder Gründen der Arbeitssicherheit vorgeschriebene besondere Arbeitskleidung (Schutzkleidung) und persönliche Schutzausrüstung unentgeltlich zur Verfügung zu stellen und instand zu halten (§ 4). Um die zahlreichen Arbeitszeitgesetzverstöße besser kontrollieren zu können, sieht § 6 GSA Fleisch eine strenge Pflicht zur Dokumentation der Arbeitszeit vor.

### Untersuchungen der Arbeitsschutzverwaltung NRW aus 2019

Die erneute Schwerpunkt-Kontrolle der Arbeitsschutzverwaltung NRW in der Fleischindustrie im Jahr 2019 („Arbeitsschutzaktion Fleischwirtschaft“) zeigte, dass auch das GSA-Fleisch nicht die erwünschte Wirkung hatte. Die Ergebnisse waren größtenteils noch bedenklicher als bei der Vorgängeruntersuchung im Jahr 2013. Die Arbeitsschutzverwaltung hatte 30 Großbetriebe und 17.000 Arbeitsplätze in der Fleischindustrie überprüft^11^ und dabei rund 8800 Rechtsverstöße festgestellt. Diese betrafen überwiegend das Arbeitszeitrecht („Arbeitsschichten von über 12 h waren keine Seltenheit“)^12^, jedoch gab es auch im Bereich des technischen Arbeitsschutzes schwerwiegende Mängel wie z. B. das Fehlen von Schutzeinrichtungen an den Arbeitsmitteln,^13^ gefährlichen Umgang mit Gefahrstoffen, abgeschlossene Notausgänge oder gefährlich abgenutzte Arbeitswerkzeuge^14^. So ist es nicht verwunderlich, dass die Anzahl der Arbeitsunfälle im Rahmen des Schlachtens, des Zerlegens und in der Verarbeitung von Fleisch weitaus höher ist als in anderen Bereichen der Nahrungsmittelindustrie.^15^ Nur zwei der untersuchten Betriebe blieben ohne gravierende Beanstandungen – beide Betriebe beschäftigten alle im Betrieb Tätigen direkt, d. h. nicht über Fremdfirmen.^16^ Momentan liegt der Anteil des Fremdpersonals in Betrieben der Fleischindustrie vielfach bei über 50 %, teilweise sogar bei 100 %. Hinzukommt, dass pro Produktionsstätte teilweise bis zu 30 verschiedene Werkvertragsunternehmen tätig sind.

### Corona-Pandemie 2020: Spezifische Situation in der Fleischwirtschaft begünstigt die Verbreitung von SARS-CoV-2

Während der Corona-Pandemie im Frühjahr und Frühsommer 2020 erwiesen sich Schlacht- und Zerlegebetriebe als sog. Hotspots für das Infektionsgeschehen. Einige Bundesländer reagierten hierauf mit eigenen Allgemeinverfügungen oder Rechtsverordnungen, die spezielle Regelungen für die Fleischindustrie vorsehen.^17^ Neben mangelndem Arbeits- und Hygieneschutz, den schlechten Arbeits- und Unterkunftsbedingungen trugen insbesondere die technischen Gegebenheiten wie die bei der Fleischzerlegung und –Verarbeitung notwendigen Kühl- und raumlufttechnischen Anlagen zu einer schnellen Verbreitung des Virus SARS-CoV‑2 bei.^18^ Diese Situation traf zahlreiche Fleischproduzenten in EU-Staaten wie Belgien, Frankreich, Irland, Spanien, Polen und den Niederlanden. Viele Fleischbetriebe haben seit Anfang April 2020 Infektionswellen unter ihren Mitarbeitern konstatiert, wobei Deutschland jedoch mit Abstand die höchste Anzahl zu verzeichnen hatte.^19^ In einer gemeinsamen Studie des Helmholtz-Zentrums für Infektionsforschung (HZI), des Universitätsklinikums Hamburg-Eppendorf (UKE) und des Heinrich-Pette-Instituts, Leibniz-Institut für Experimentelle Virologie (HPI) wurden die Ursprünge des ersten SARS-CoV-2-Ausbruchs im Mai 2020 bei Tönnies in Rheda-Wiedenbrück, dem größten Fleischverarbeitungskomplex Deutschlands, untersucht. Die Ergebnisse zeigen, dass das Virus von einem einzigen Beschäftigten ausging und auf mehrere Personen in einem Umkreis von mehr als acht Metern übertragen wurde. Die Ergebnisse weisen darauf hin, dass die spezifischen Bedingungen des Zerlegebetriebes (niedrige Temperatur von 10 °C, geringe Frischluftzufuhr und eine konstante Luftumwälzung durch die Klimaanlage in Verbindung mit anstrengender körperlicher Arbeit) eine Aerosolübertragung des Virus auch über größere Entfernungen begünstigt haben.

## Kooperation im Arbeitsschutz bei Fremdarbeitnehmer-Konstellationen: Kein Erfolgsmodell in der Fleischindustrie

Bereits 2013 berichtete die Arbeitsschutzverwaltung Nordrhein-Westfalen in ihrem Jahresbericht, dass in der Mehrzahl der Betriebe in der Fleischwirtschaft Werkvertragsunternehmen eingesetzt werden, die wiederum nicht selten mit entsandten Arbeitnehmern tätig sind. Der „Branchenmonitor Schlachten und Fleischverarbeitung“ der Hans-Böckler-Stiftung von Dezember 2019 zählt 50–90 % der Beschäftigten im Schlachtbereich zu den Werkvertragsbeschäftigten, Tendenz steigend.^20^ Werkvertragsunternehmen sind seit geraumer Zeit nicht mehr nur für periphere Tätigkeitsbereiche wie Reinigung- und Bewachungsdienste, die Externalisierung betrifft vielmehr auch die Kerntätigkeit der Fleischwirtschaft, das Zerlegen und Verarbeiten von Fleisch. Diese Konstellation stellt besondere Anforderungen an den Arbeitsschutz und die Klärung der jeweiligen Verantwortlichkeit.^21^

### Zuständigkeit für Arbeitsschutz in Fremdarbeitnehmer-Konstellationen – Kooperation ist Pflicht

Für Entsendungen sieht das EU-Recht bereits seit 1996 verpflichtende Schutzbestimmungen unabhängig von der Dauer der Entsendung vor,^22^ die – wenn auch mit spezifischen Defiziten – im Arbeitnehmerentsendegesetz ins deutsche Recht umgesetzt wurden. Nach dem Entsenderecht ist der Beschäftigungsstaat – also Deutschland – verpflichtet, die Einhaltung des in der Entsende-Richtlinie definierten harten Kerns an Schutzvorschriften des Aufnahmemitgliedstaates zu garantieren. Zu diesem Kern gehört nach Art. 3 der Entsende-RL^23^ somit das deutsche Arbeitsschutz- (Sicherheit, Gesundheitsschutz und Hygiene am Arbeitsplatz) und Arbeitszeitrecht (Höchstarbeitszeiten und Mindestruhezeiten). Im AEntG sind bisher keine spezifischen Kontrollvorschriften normiert worden. Ebenso wie in anderen Fremdfirmen-Konstellationen stellen sich typische Herausforderungen für den Werkunternehmer in seiner Rolle als Arbeitgeber, da die Beschäftigten ihre Arbeitsleistung im Einsatzbetrieb erbringen und somit den dortigen Gefährdungen und Gegebenheiten ausgesetzt sind.^24^

Durch die Erbringung der Arbeitsleistung in einem Fremdbetrieb gestaltet sich oftmals bereits die Gefährdungsbeurteilung schwierig, insbesondere, da vielfach auf Maschinen und Produktionsmittel des Auftraggebers zurückgegriffen wird.^25^ Eine enge Kooperation zwischen Auftraggeber und (oftmals ausländischem) Werkvertragsunternehmer ist somit unabdingbar; diese Kooperationspflicht, die in § 8 ArbSchG^26^ statuiert wird, erstreckt sich auch auf entsandte Arbeitnehmer.^27^ Nach § 8 Abs. 1 ArbSchG haben Arbeitgeber an Arbeitsplätzen, an welchen mehrere Beschäftigte verschiedener Arbeitgeber tätig sind, bei der Durchführung der Sicherheits- und Gesundheitsschutzbestimmungen zusammenzuarbeiten. Soweit dies für die Sicherheit und den Gesundheitsschutz der Beschäftigten bei der Arbeit erforderlich ist, haben die Arbeitgeber je nach Art der Tätigkeiten insbesondere sich gegenseitig und ihre Beschäftigten über die mit den Arbeiten verbundenen Gefahren für Sicherheit und Gesundheit der Beschäftigten zu unterrichten und Maßnahmen zur Verhütung dieser Gefahren abzustimmen. In der Fleischwirtschaft ist eine intensive Kooperation ob der vielfältigen, der Art der Tätigkeit immanenten Gefahren zwingend, wird in der Praxis jedoch kaum praktiziert.^28^ Einschlägige Kooperations- und Überwachungspflichten sind im deutschen Recht – auch wieder in Konkretisierung des Unionsrechts – auch in §§ 15 GefStoffV, 13 BetrSichV normiert. Bei diesen gesundheitsgefährlichen Tätigkeiten muss eine Koordination zwischen den verschiedenen im Betrieb tätigen Arbeitgebern erfolgen. Die Aufsicht hat sich auch darauf zu erstrecken, dass geeignete Koordinatoren bestellt worden sind. Nach den bisherigen Berichten ist diese Aufsicht kaum erfolgt.

### Mangelnde Kooperation und Überwachung – ein EU-weites Problem

Die deutsche Situation ist allerdings kein Ausnahmefall. Bereits 2009 hat die Kommission einen Forschungsauftrag an juristische Expertinnen und Experten aus den meisten Mitgliedsstaaten gegeben, mit dem eine vergleichende Studie über die rechtlichen Aspekte der Entsendung von Arbeitnehmern im Netzwerk des Unionsrechts zu erstellen war. Diese Studie ist unter der Leitung von Aukje van Hoek und Mijke Houwerzijl im März 2011 veröffentlicht worden.^29^ Eine weitere ergänzende Studie ist dann im Herbst 2011 vorgelegt worden.^30^ Beide Studien erfassen zusammen fast alle Mitgliedsstaaten der Union. In den eher empirisch orientierten Teilen der Studien wird sehr deutlich herausgearbeitet, dass von einer effektiven Überwachung und Rechtsdurchsetzung in der Mehrzahl der Mitgliedsstaaten nicht die Rede sein kann. Die Autorinnen und Autoren haben dabei auch verdeutlicht, dass die Kooperations- und Überwachungspflichten nach Art. 6, 10 und 12 der RL 89/391/EWG, die in Deutschland vor allem durch § 8 ArbSchG umgesetzt worden sind,^31^ gerade auch die Situation entsandter Arbeitnehmer betreffen,^32^ aber kaum beachtet werden.

Angesichts der deutlichen empirischen Defizite, die die Kommission durch ihre Forschungsarbeiten festgestellt hatte, ist bereits 2014 eine weitere Richtlinie beschlossen worden, die RL 2014/67/EU. Diese „Durchsetzungsrichtlinie“ schafft keine neuen materiell-rechtlichen Pflichten, sondern soll sicherstellen, dass die bisherige unzureichende Rechtsdurchsetzung verbessert wird. Diese Richtlinie ist in Deutschland nicht umgesetzt worden^33^ Gerade für Arbeitsschutzpflichten sind in der Durchsetzungsrichtlinie konkrete Meldepflichten vorgeschrieben worden. In einer aktuellen unionsrechtlichen Kontrolle ist festgestellt worden, dass die deutschen Arbeitsschutzbehörden noch nicht einmal regelmäßige Berichte über Arbeitsunfälle entsandter Arbeitnehmer erhalten^34^. Insoweit ist hier eine Korrektur dringend erforderlich, die auch im neuen Entsenderecht sowie Arbeitsschutzkontrollgesetz bisher noch nicht erfolgt ist.

### Zuspitzung der Arbeitsschutzdefizite in Pandemie-Zeiten

Wie die bereits erwähnte Untersuchung des COVID-19-Ausbruchs bei Tönnies in Rheda-Wiedenbrück gezeigt hat, spielen raumlufttechnische Anlagen (RLT-Anlagen) eine kritische Rolle beim Infektions- und somit auch Arbeitsschutz. An raumlufttechnische Anlagen sind gerade in Pandemiezeiten erhöhte Anforderungen zu stellen, weil bei unzureichender Wartung spezifische Gefährdungen auftreten können. Diese Anforderungen sind bereits in der allgemeinen Arbeitsstätten-Richtlinie ASR A 3.6 „Lüftung“ am Stand der Technik orientiert worden^35^. In der Technischen Regel Corona vom 11.08.2020 ist die Bedeutung der Kontrolle der RLT-Anlagen zusätzlich hervorgehoben worden. Hier ist in der Gefährdungsbeurteilung zu überprüfen, ob die in der VDI-Richtlinie 6022 verlangten Hygienekontrollen ordnungsgemäß durchgeführt und dokumentiert worden sind. Besonders in Fremdfirmenkonstellationen ist eine Gewährleistung des Infektionsschutzes durch den Werkvertragsunternehmer – allein schon durch die Eigentumsverhältnisse an Arbeitsstätte und Produktionsmitteln – allerdings kaum möglich.

### Der Betriebsinhaber als Schlüsselfigur für den Arbeitsschutz

Gerade die empirischen und arbeitswissenschaftlichen Untersuchungen zeigen, dass bei dem Konstrukt der Werkvertragsgeflechte vor allem in der Fleischindustrie das Leitbild des § 8 ArbSchG nicht ausreicht. Es handelt sich hier um eine Kooperationspflicht zwischen zwei Unternehmern, die als gleichgewichtig vorgestellt werden. Wenn jedoch ein Betriebsinhaber bis zu 20 Subunternehmern in seinem Betrieb Aufgaben überträgt, dann besteht ein eindeutiges Machtungleichgewicht. Das Leitbild der Kooperationspflichten trägt nicht. Deutlich zeigt dies die Situation der raumlufttechnischen Anlagen, die als virenförderliche Instrumente inzwischen identifiziert worden sind. Bereits die Vorstellung, der jeweilige rumänische oder bulgarische Werkunternehmer könnte nach § 8 Abs. 1 ArbSchG den deutschen Betriebsinhaber in einem Vertrag verpflichten, die bisherigen raumlufttechnischen Anlagen umzubauen und regelmäßige Hygienekontrollen durchzuführen, damit die Gesundheit seiner Werkvertragsbeschäftigten gesichert wird, ist so irreal, dass hier von Kooperationsregeln kein Erfolg erwartet werden kann. Insofern sind die 2011 ermittelten Ergebnisse über die unionsweiten Defizite bei der Umsetzung der Kooperationspflichten ein wichtiges Indiz, dass dieses Instrument bei Werkverträgen im Kernbereich der Tätigkeiten des Betriebsinhabers keinen ausreichenden Gesundheitsschutz vermitteln kann.

Vielmehr handelt es sich hier um Situationen, in denen Organisationspflichten des Betriebsinhabers erforderlich sind und letztlich durch die Aufsicht kontrolliert werden müssen. In einem wichtigen Beitrag haben Christian Paschke und Christine Schulze-Doll bereits 2016 herausgearbeitet, dass bei einer Integration der Werkvertragsbeschäftigten in betriebliche Gefährdungen des Betriebs diesem Inhaber zusätzliche Schutzpflichten zugeordnet werden müssen^36^. Als Parallelwertung haben sie dazu auf die Regeln der Leiharbeit hingewiesen. In Umsetzung der RL 91/383/EWG ist durch § 11 Abs. 6 AÜG der jeweilige Entleiher verpflichtet worden, auch die Arbeitsschutzpflichten zugunsten der Arbeitnehmer des Verleihers zu beachten^37^. Diese Rollenverteilung ist durch das Bundesarbeitsgericht ausdrücklich anerkannt worden.^38^ Diese Parallele trägt hier, weil in der Praxis nicht selten Werkverträge zu beobachten sind, die sich letztlich als verdeckte und unzulässige Arbeitnehmerüberlassung erweisen.

Zur Abgrenzung zwischen Arbeitnehmerüberlassung und Werkverträgen verlangt das BAG seit 25 Jahren, dass der Werkunternehmer in der Lage ist, seinen arbeitsrechtlichen Pflichten durch eine geeignete und eigenständige Organisation nachzukommen^39^. Anhand eines Beispiels aus der Fleischwirtschaft hat das LAG Berlin-Brandenburg 2012 entschieden, dass bei nicht hinreichend und eigenständig abgrenzten Arbeitsaufgaben ein Scheinwerkvertrag vorliegt.^40^ Dies entspricht ausführlichen empirischen Untersuchungen der TU Chemnitz, die gerade bei Onsite-Werkverträgen auf dem Betriebsgelände und im Kernbereich des Bestellers eine ausgedehnte Grauzone mit nicht hinreichend abgegrenzten Werkvertragspflichten festgestellt hatten.^41^

Wer zusätzliche anschauliche Sachverhalte suchen will, wird bei strafrechtlichen Entscheidungen fündig. In einem erst vor kurzem ergangenen Strafurteil ist die vor mehr als zehn Jahren realisierte Durchführung von Scheinwerkverträgen von fast 1000 bulgarischen Arbeitnehmern inzwischen rechtskräftig als strafbares Handeln qualifiziert worden.^42^ Arbeitsrechtlich bedeuten solche Scheinwerkverträge eine Form verbotener Arbeitnehmerüberlassung, die in Anwendung von § 10 AÜG zu einer endgültigen Anstellung der Werkvertragsbeschäftigten beim Betriebsinhaber führen.^43^

Unter Arbeitsschutzgesichtspunkten ist es nicht ausreichend, den Gesundheitsschutz nur in Fällen strafbarer Scheinwerkverträge zu gewährleisten. Der Gesundheitsschutz ist Korrelat einer vor allem arbeitsstättenbezogenen Gefährdung; diese Gefährdung aber ergibt sich aus einer Organisation des Zusammenwirkens verschiedener Beteiligter, die nur durch den Betriebsinhaber effektiv gesteuert werden kann. Aus diesem Grund ist die aktuelle Rechtspolitik auf dem richtigen Weg.

## Lebensmittelsicherheit verlangt effektiven Arbeitsschutz

Dieser Weg wird bestätigt durch das Recht der Lebensmittelsicherheit. Insbesondere in der Lebensmittelindustrie ist es für einen effektiven Arbeitsschutz und die Lebensmittelsicherheit notwendig, Hygiene- und Arbeitsschutzbestimmungen im Zusammenhang zu sehen, denn: die Einhaltung der Arbeits- und Hygienebestimmungen ist in diesem Fall auch vorbeugender Verbraucherschutz. Dies gilt umso mehr in Bereichen der Tierprodukteverarbeitung: eine effektive Lebensmittelhygiene kann ohne funktionierenden Arbeitsschutz kaum gewährleistet werden – und umgekehrt.

Auch die Bestimmungen des Lebensmittelhygienerechts stützen das Gebot, in den Kernbereichen der Fleischwirtschaft mit eigenen Betriebsangestellten zu arbeiten. In der Diskussion um die Arbeitsbedingungen in der Fleischindustrie wird das EU-Verbraucherrecht – und insbesondere das Lebensmittelhygienerecht – bisher weitgehend außen vor gelassen. Doch gerade im Zusammenhang mit der Herstellung und Verarbeitung von Lebensmitteln ist ein Blick in das diesbezügliche Sekundärrecht im Grunde unumgänglich. Auch hier kommt dem Betriebsinhaber eine entscheidende Schlüsselrolle zu, da regelmäßig nur dieser die betrieblichen Gegebenheiten wie technische Anlagen und Produktionsmittel verantworten kann.

### Das Hygienerecht der EU

Das EU-Lebensmittelhygienerecht verfolgt nach seiner Reformierung in den Jahren 2004 und 2005 ein integriertes Konzept vom Erzeuger zum Verbraucher unter Einbeziehung aller Stationen der Lebensmittelkette von der Futtermittelerzeugung bis zum Einzelhandelsverkauf.^44^ Die für die Fleischwirtschaft einschlägigen Verordnungen Nr. 852/2004/EG^45^, Nr. 853/2004/EG^46^ und Nr. 854/2004/EG^47^ (sog. Hygienepaket) bestimmen Hygienevorschriften, die auf allen Produktions- und Verarbeitungsstufen einzuhalten sind. Dabei handelt es sich u. a. um Vorschriften für Betriebsstätten, Räume, Ausrüstungen, zur Wasserversorgung, zur Personalhygiene, zum Umgang mit Lebensmitteln im Betrieb und zur Personalschulung (Anhang II VO Nr. 852/2004/EG ergänzt durch Anhang III VO Nr. 853/2004/EG). Zentral für die Lebensmittelhygiene ist eine Gefahranalyse und Kontrolle, die auf den sog. HACCP-Grundsätzen beruht.^48^ Hierdurch sollen spezifische Gesundheitsgefahren, die mit einem bestimmten Lebensmittel verbunden sein können, verhindert bzw. beherrscht werden.^49^ Die primäre rechtliche Verantwortung für die Gewährleistung der Lebensmittelsicherheit nach den Verordnungen obliegt dem Lebensmittelunternehmer. Die Verordnungen gelten gemäß Art. 288 Abs. 2 unmittelbar in den Mitgliedstaaten.^50^ Ergänzt werden sie durch die nationalen Bestimmungen der Lebensmittelhygiene-Verordnung und der Tierischen Lebensmittelhygiene-Verordnung.

### Zulassungspflicht für Betriebe der Fleischwirtschaft nach VO Nr. 853/2004/EG – der Betriebsinhaber (auch hier) als Schlüsselfigur

Die EU-Verordnung Nr. 853/2004 mit spezifischen Hygienevorschriften für Lebensmittel tierischen Ursprungs sieht in Artikel 4 Abs. 2 vor, dass Betriebe, die mit Erzeugnissen tierischen Ursprungs umgehen, erst nach Zulassung durch die zuständige Behörde ihre Tätigkeit aufnehmen dürfen. Zu den Betrieben i. S. d. Verordnung zählen Schlachthäuser, Zerlegungsbetriebe, Fleischzubereitungs- und Fleischerzeugnisseherstellungsbetriebe.^51^ Für eine Zulassung muss der Lebensmittelunternehmer neben einer Besichtigung der Anlagen durch die zuständige Überwachungsbehörde den Nachweis über die Einhaltung der einschlägigen Anforderungen nach den VO Nr. 852/2004/EG und Nr. 853/2004/EG erbringen. Infolgedessen ist der Lebensmittelunternehmer für die Einhaltung der Hygienevorschriften verantwortlich, um so den Verbraucherschutz zu gewährleisten. In der in der deutschen Fleischwirtschaft vorherrschenden Konstellation der Vergabe von Werkverträgen für Tätigkeiten wie das Schlachten, Zerlegen und Verarbeiten von Fleisch und einer teilweisen Quote von 100 % Fremdarbeitnehmern im Betrieb scheint eine solche Sicherstellung des Verbraucherschutzes jedoch schwerlich realisierbar. Der Verbraucherschutz verlangt, dass auch der Arbeits- und Gesundheitsschutz der Beschäftigten gewährleistet ist, um Hygienemängel im Produktionsprozess zu vermeiden. Dies gilt insbesondere in Zeiten einer Pandemie. Wie die eingangs dargestellten Untersuchungen ergeben haben, gibt es in der Fleischwirtschaft im Arbeitsschutz- und Arbeitssicherheitsbereich eklatante Mängel, die zu einem erheblichen Teil auf die Fremdfirmenkonstruktion zurückzuführen sind. Der Lebensmittelunternehmer kann insofern die essentiellen Kriterien der Zulassung kaum dauerhaft erfüllen, denn eine Delegation der Verantwortung auf den Werkvertragsnehmer, der selbst gerade nicht über eine behördliche Zulassung nach § 9 Tier-LMHV bzw. Art. 4 Abs. 2 VO Nr. 853/2004/EG verfügt, scheint mit der Systematik und dem Sinn und Zweck der Verordnung kaum vereinbar. Es ist eindeutig, dass als zulassungsbedürftiger Betrieb hier der Auftraggeber und Betriebsinhaber und nicht die zahlreichen Subunternehmer zu qualifizieren ist.

## Der Arbeitsschutzkontrollgesetz-Entwurf der Bundesregierung – erste Schritte in die richtige Richtung

Zentrales Vorhaben ist, das bisher sehr verbreitete problematische Werkvertrags-Konstrukt in der Fleischindustrie durch ein Direktanstellungsgebot in den „Kernbereichen“ zu unterbinden. Betriebsorganisation und Personalverantwortung sollen somit wieder allein beim Inhaber liegen. Werkverträge sollen ab 01.01.2021, Leiharbeit ab 01.04.2021 nicht mehr möglich sein. Kernbereich meint gemäß § 6a GSA Fleisch-Entwurf hierbei die Schlachtung, Zerlegung und Verarbeitung von Fleisch. Der Inhaber ist dann für alle Beschäftigten in seinem Kerngeschäft zuständig. Ausgenommen hiervon sind nur Unternehmen des Fleischerhandwerks mit bis zu 49 im Betrieb tätigen Personen. Der Gesetzesentwurf operiert mit dem Begriff „Inhaber“ und definiert diesen als „wer über die Nutzung der Betriebsmittel und den Einsatz des Personals entscheidet. Wenn aufgrund der räumlichen oder funktionalen Einbindung des Betriebes in eine übergreifende Organisation die Arbeitsabläufe in dem Betrieb inhaltlich oder zeitlich im Wesentlichen vorgegeben sind, ist Inhaber, wer die übergreifende Organisation führt.“ Somit ist die gemeinsame Führung eines Betriebes oder eine übergreifende Organisation durch mehrere Unternehmen unzulässig. Dieser Entwurf hat von der Mehrzahl der Parteien und der Bundesländer eine Unterstützung erfahren. Die BDA spricht in ihrer Stellungnahme dagegen von einer „Schlachtung der Unternehmerfreiheit“.^52^ Wenn man die Zahl von deutlich mehr als 1000 mit SARS-CoV‑2 infizierten Werkvertragsbeschäftigten allein in der Fleischwirtschaftet berücksichtigt, dann ist hier deren Gesundheitsrecht „geschlachtet“ worden. Die staatlichen Schutzpflichten verlangen daher effektive Schutzmaßnahmen. In der Gerichtspraxis einiger unserer Nachbarstaaten ist während der Corona-Pandemie die Notwendigkeit effektiver Schutzpflichten auf das europäische Grundrecht in Art. 31 EU-GRCh gestützt worden. Nach der deutschen Rechtssystematik ist dieses Grundrecht bei der Auslegung der bisherigen Vorschriften und vor allem bei der Konstruktion neuer Vorschriften zu beachten. Nachdem der Europäische Gerichtshof in den letzten Jahren mehrfach die Mitgliedsstaaten ermahnt hat, dieses Grundrecht effektiv zur Geltung zu bringen, ergibt sich daraus auch eine verfassungsrechtliche Schutzpflicht^53^. Das Arbeitsschutzkontrollgesetz enthält dazu erste Ansätze für eine Branche, bei der besonders dringlich Maßnahmen erforderlich sind.^54^ Im Rahmen einer Verbesserung der Arbeitsschutzkontrollen werden sich daraus auch Konsequenzen für andere Branchen und Konstellationen ergeben.

Letztlich dient der Gesetzentwurf der Zuordnung klarer Verantwortlichkeiten, so dass zugleich die Rechtsdurchsetzung und Kontrolle realisiert werden kann. „Ein Verbot von Werkverträgen und Arbeitnehmerüberlassung bedeutet, dass derjenige, der das Arbeitsumfeld, die Prozesse im Betrieb und den Umfang der Arbeit bestimmt, allein auch für die Einhaltung der arbeits- und arbeitsschutzrechtlichen Standards bezüglich aller im Schlachthof eingesetzten Arbeitnehmerinnen und Arbeitnehmer Sorge zu tragen hat“^55^.

Damit findet diese Regelung eine bemerkenswerte Parallele zum Verbot der Arbeitnehmerüberlassung im Baugewerbe, das zunächst in § 12a AFG und jetzt in § 1b AÜG normiert ist. Dieses Verbot ist vom Bundesverfassungsgericht 1987 bestätigt worden. Der damals verfolgte Zweck ist vom Gericht damals so umschrieben worden:

„Die Wiederherstellung der gestörten Ordnung auf dem Teilarbeitsmarkt des Baugewerbes mit dem Ziel der Sicherung eines geordneten Arbeitsmarktes und einer stabilen arbeits- und sozialversicherungsrechtlichen Situation abhängig Beschäftigter ist ein hervorragend wichtiges Gemeinschaftsgut“^56^.

Die bisherige Werkvertragspraxis hat den Arbeitsschutz so intensiv zerlegt, dass er nicht mehr realisiert werden kann. Das Verbot der Werkverträge im Kernbereich der Fleischwirtschaft und die Zuordnung zu einem Betriebsinhaber schafft klare Verantwortlichkeiten und damit in dieser Branche einen integrativen Arbeitsschutz^57^.

Am 5. Oktober 2020 fand eine Anhörung von Sachverständigen und Verbänden im Bundestagsausschuss für Arbeit und Soziales statt. Sämtliche Stellungnahmen sind auf der Website des Ausschusses dokumentiert (Drucksache 19(11)778). Strittig blieben letztlich die Definition des Kernbereichs und das Verbot von Leihverträgen. Deswegen ist das Arbeitsschutzkontrollgesetz am 26. Oktober von der Tagesordnung abgesetzt; zum Redaktionsschluss der Zeitschrift am 16. November war noch keine endgültige Lösung gefunden worden.

## Footnotes

1 Kontrollen der Arbeitsschutzverwaltung NRW haben 2020 bereits knapp 1900 Beanstandungen festgestellt – vom Fehlen einfacher Hygienemaßnahmen über Schimmelpilz- und Ungezieferbefall, undichten Dächern, katastrophale Sanitäreinrichtungen bis zu Objekten mit Einsturzgefahr. Gesetzesentwurf, S. 3.

2 Gesetzesentwurf für ein Gesetz zur Verbesserung des Vollzugs im Arbeitsschutz (Arbeitsschutzkontrollgesetz) vom 07.08.2020 – BR-Drucksache 426/20.

3 vgl. z. B. das Gesetz zur Sicherung von Arbeitnehmerrechten in der Fleischwirtschaft, GSA Fleisch, vom 17.07.2017, BGBl. I S. 2541.

4 Vgl. die Vielzahl von Aufsätzen und Medienbeiträgen zur Situation in der Fleischindustrie z. B.: Tornau, Für halb so viel Lohn, Mitbestimmung 2017, S. 20; Mayert, Menschengerechte Arbeit in der Fleischindustrie?, ZfArbWiss 2018, S. 208; Weinkopf, Arbeitsbedingungen in der Fleischwirtschaft im Vergleich, ZfArbWiss 2018, S. 213; Kohte, Gesundheitsschutz in der Grauzone? – Arbeitsbedingungenentsandter Beschäftigter, FS für Eichenhofer, 2015, S. 313; Antwort der Bundesregierung auf die kleine Anfrage der Fraktion Bündnis 90/Die Grünen v. 23.12.2010: BT-Drs. 17/4341; Doelfs, Deutsche Fleischindustrie – Europas Schmuddelecke, Magazin Mitbestimmung 2016, 62 (6), S. 32–34; Knigge, Vom Kampf gegen die Ausbeutung in der Fleischindustrie, Deutschlandfunk v. 27.09.2016, abrufbar unter: https://www.deutschlandfunk.de/missstaende-bei-leiharbeitern-vom-kampf-gegen-ausbeutung-in.724.de.html?dram:article_id=367021 (12.08.2020).

5 Arbeitsschutz in Nordrhein-Westfalen, Jahresbericht 2013, S. 24.

6 Arbeitsschutz in Nordrhein-Westfalen, Jahresbericht 2013, S. 22.

7 BGBl. I S. 538.

8 vgl. Küppers/Nothelle-Wildfeuer ZfArbWiss 2018, 204, 206; Weinkopf, ZfArbWiss 2018, 213, 214.

9 Als Art. 30 des Gesetzes zur Änderung des Bundesversorgungsgesetzes und anderer Vorschriften. Siehe hierzu Düwell, jurisPK-ArbR 23/2017, Anm. 1.

10 Jetzt § 6 Abs. 9 AEntG 2020, BGBl. I 2020, 1657, 1658.

11 Anzumerken ist, dass die Zahl der von den Arbeitsschutzbehörden durchgeführten Betriebsbesichtigungen allerdings seit Jahren rückläufig ist, Gesetzesentwurf, S. 1.; vgl. Kohte, WSI-Mitteilungen 3/2015, S. 170 ff.

12 Presseinformation – 856/10/2019 der Landesregierung Nordrhein-Westfalen v. 16.10.2019, S. 2.

13 Gesetzesentwurf, S. 2.

14 Presseinformation – 856/10/2019 der Landesregierung Nordrhein-Westfalen v. 16.10.2019, S. 2.

15 Die Häufigkeit lag bereits 2010 bei fast dem dreifachen Wert vieler anderer Branchen wie die Antwort der Bundesregierung auf die kleine Anfrage der Fraktion Bündnis 90/Die Grünen v. 23.12.2010 zeigt: BT-Drs. 17/4341, S. 8.

16 Presseinformation – 856/10/2019 der Landesregierung Nordrhein-Westfalen v. 16.10.2019, S. 2.

17 Vgl. z. B. Allgemeinverfügung des Ministeriums für Arbeit, Gesundheit und Soziales des Landes Nordrhein-Westfalen zur Vermeidung weiteren Infektionsgeschehens in Großbetrieben der Fleischwirtschaft; Corona-Verordnung Schlachtbetriebe und Fleischverarbeitung Baden-Württemberg.

18 Thomas Günther, Manja Czech-Sioli, Daniela Indenbirken, Alexis Robitailles, Peter Tenhaken, Martin Exner, Matthias Ottinger, Nicole Fischer, Adam Grundhoff, Melanie M. Brinkmann: Investigation of a superspreading event preceding the largest meat processing plant-related SARS-Coronavirus 2 outbreak in Germany, SSRN, 2020.

19 Nach Deutschland war in der EU insbesondere die irische Fleischindustrie von Ansteckungen auf Schlachthöfen betroffen – in 19 Betrieben infizierten sich insgesamt 950 Arbeiter mit dem Virus, in einigen Fällen erkrankte ein Viertel der Beschäftigten. Nack, Europäische Fleischindustrie als Corona-Infektionsherd, Deutsche Welle v. 28.06.2020, abrufbar unter: https://www.dw.com/de/europ%C3%A4ische-fleischindustrie-als-corona-infektionsherd/a-53955048 (12.08.2020).

20 Branchenmonitor Schlachten und Fleischverarbeitung, herausgegeben von der Hans-Böckler-Stiftung, Dezember 2019, S. 16.

21 Zur Verteilung der Arbeitsschutzpflichten bei Fremdfirmeneinsatz: Paschke/Schulze-Doll, Arbeitsschutz im Rahmen von Werkverträgen unter besonderer Berücksichtigung der Mitbestimmung des Betriebsrats, in: Festschrift für Kohte, 2016, S. 493 ff.

22 Richtlinie 96/71/EG des Europäischen Parlaments und des Rates vom 16. Dezember 1996 über die Entsendung von Arbeitnehmern im Rahmen der Erbringung von Dienstleistungen geändert durch Richtlinie (EU) 2018/957 des Europäischen Parlaments und des Rates vom 28. Juni 2018 zur Änderung der Richtlinie 96/71/EG über die Entsendung von Arbeitnehmern im Rahmen der Erbringung von Dienstleistungen, flankiert durch Richtlinie 2014/67/EU des Europäischen Parlaments und des Rates vom 15. Mai 2014 zur Durchsetzung der Richtlinie 96/71/EG über die Entsendung von Arbeitnehmern im Rahmen der Erbringung von Dienstleistungen (Durchsetzungs-Richtlinie).

23 sowohl in der RL 96/71/EG als auch in der RL 2018/957/EU.

24 Wiebauer, ZfA 2014, S. 29, 31.

25 Paschke/Schulze-Doll, Arbeitsschutz im Rahmen von Werkverträgen unter besonderer Berücksichtigung der Mitbestimmung des Betriebsrats, in: Festschrift für Kohte, 2016, S. 493, 494 ff.

26 Zu den unionsrechtlichen Vorgaben aus den Art. 6, 10 und 12 der RL 89/391/EWG, welche in Art. 8 ArbSchG umgesetzt sind, siehe Julius, Arbeitsschutz und Fremdfirmenbeschäftigung, 2004, S. 48 ff, 150 ff.

27 Kohte, FS Eichenhofer, S. 313, 318; HK-ArbSchR/Julius § 8 ArbSchG Rn. 13.

28 Kohte, FS Eichenhofer, S. 313, 318 f.

29 Van Houk/Houwerzijl, Comparative study on the legal aspects of the posting of workers in the framework of the provision of services in the European Union, März 2011.

30 Van Houk/Houwerzijl, Complementary study on the legal aspects of the posting of workers in the framework of the provision of services in the European Union, November 2011; vgl. Houwerzijl, AuR 2013, 342 ff.

31 Julius, Arbeitsschutz und Fremdfirmenbeschäftigung, 2004, S. 48 ff, 150 ff; vgl. MHdBArbR/Kohte, 4. Aufl. 2018 § 173 Rn. 25.

32 Bremer, Arbeitsschutz im Baubereich, 2007, S. 20.

33 Zur Kritik Kohte, Festschrift für Eichenhofer S. 313, 323 ff.

34 SLIC –Revision 2017 des staatliche Arbeitsschutzsystems der Bundesrepublik Deutschland, www.lasi-info.com/publikationen/internationales-slic.

35 Pernack/Tannenhauer/Pangert, Arbeitsstätten, 9. Aufl. 2017, S. 348 ff.

36 Paschke/Schulze-Doll, FS Kohte, 2016, S. 493 ff.

37 Bücker/Feldhoff/Kohte, Vom Arbeitsschutz zur Arbeitsumwelt, 1994 Rn. 272 ff.

38 BAG NZA 2016, 1420.

39 BAG NZA 1995, 572; Ulber/Ulber AÜG, 3. Aufl. 2020 Einl. Rn 42 ff.

40 LAG B-B 12.12.2012 – 15 Sa 1217/12, BB 2013, 1020, zustimmend ErfK/Wank 20. Aufl. 2020 AÜG § 1 Rn. 29.

41 Hertwig/Kirsch/Wirth AuR 2016, 141 ff und ausführlich dies., Werkverträge im Betrieb, Boeckler study 300, 2015, S. 98 ff.

42 BGH 07.03.2019 – 3 StR 192/18, NStZ 2020, 170.

43 ErfK/Wank § 10 AÜG Rn. 7.

44 Einen fundierten Überblick gibt Murmann in Streinz/Kraus, Lebensmittelrechts-Handbuch, Stand Juli 2019, Rn 126 ff.

45 VERORDNUNG (EG) Nr. 852/2004 DES EUROPÄISCHEN PARLAMENTS UND DES RATES vom 29. April 2004 über Lebensmittelhygiene, ABl. 2004 L 139 S. 1.

46 VERORDNUNG (EG) NR. 853/2004 DES EUROPÄISCHEN PARLAMENTS UND DES RATES vom 29. April 2004 mit spezifischen Hygienevorschriften für Lebensmittel tierischen Ursprungs, ABl. 2004 L 139 S. 55.

47 VERORDNUNG (EG) Nr. 854/2004 DES EUROPÄISCHEN PARLAMENTS UND DES RATES vom 29. April 2004 mit besonderen Verfahrensvorschriften für die amtliche Überwachung von zum menschlichen Verzehr bestimmten Erzeugnissen tierischen Ursprungs, ABl. 2004 L 139 S. 206.

48 Art. 5 VO Nr. 852/2004/EG. Gefahranalyse und kritische Kontrollpunkte (engl. *hazard analysis and critical control points*, abgekürzt HACCP) ist ein strukturiertes, auf präventive Maßnahmen ausgerichtetes Qualitätswerkzeug und dient der Lebensmittelsicherheit.

49 Murmann in Streinz/Kraus, Rn. 127.

50 Zu den möglichen Abweichungen vom EU-Recht durch nationales Recht siehe Murmann in Streinz/Kraus, Rn 133a ff.

51 Vgl. den Leitfaden der EU-Kommission für die Umsetzung einzelner Bestimmungen der Verordnung (EG) Nr. 853 /2004 v. 21. Dezember 2005, Anhang IV.

52 So auch Wolfgang Kleinebrink, Entwurf eines Arbeitsschutzkontrollgesetzes – oder: Wenn die Unternehmensfreiheit „geschlachtet“ wird, ArbRB-Blog, abrufbar unter https://www.arbrb.de/blog/2020/07/31/entwurf-eines-arbeitsschutzkontrollgesetzes-oder-wenn-die-unternehmerfreiheit-geschlachtet-wird/, 05.08.2020; siehe auch den Kommentar in der Frankfurter Allgemeinen Zeitung v. 29.07.2020 „Schlachtfest in der Fleischindustrie“, abrufbar unter: https://www.faz.net/aktuell/wirtschaft/schlachtfest-in-der-fleischindustrie-kommentar-16880300.html, 05.08.2020.

53 HK-ArbSchR, 2. Aufl. 2018 – Lörcher, Grundrechte Rn. 43 ff und Kohte, Unionsrecht Rn. 38.

54 Der Gesetzesentwurf sieht vor, dass die Arbeitsschutzbehörden der Länder eine Mindestanzahl an Betrieben pro Jahr zu besichtigen haben (einheitliche Mindestkontrollquote von 5 % der im Land vorhandenen Betriebe ab 2026). Eine Bundesaufsicht soll die Umsetzung der gesetzlichen Regelung absichern (Monitoring der Arbeitsschutzaufsicht durch eine einzurichtende Bundesfachstelle bei der BAuA).

55 BR-Drs 426/20, S. 28.

56 BVerfG v. 06.10.1987 – 1 BvR 1086/82 u. a., BVerfGE 77, 84 – juris, Rn. 77.

57 so auch Olaf Deinert, Kurzgutachten: Womit kann begründet werden, dass Werkverträge und Arbeitnehmerüberlassung nur in der Fleischindustrie verboten werden können? Gutachten im Auftrag des Ministeriums für Arbeit, Gesundheit und Soziales des Landes Nordrhein-Westfalen v. 15.07.2020, abrufbar unter: https://www.mags.nrw/gutachten-werkvertraege-fleischindustrie.

